# Investigating the effects and mechanisms of Erchen Decoction in the treatment of colorectal cancer by network pharmacology and experimental validation

**DOI:** 10.3389/fphar.2022.1000639

**Published:** 2022-10-13

**Authors:** Yanfei Shao, Jingxian Chen, Yujie Hu, Yuan Wu, Hualin Zeng, Shuying Lin, Qiying Lai, Xiaodong Fan, Xueliang Zhou, Minhua Zheng, Bizhen Gao, Jing Sun

**Affiliations:** ^1^ Department of General Surgery, Ruijin Hospital, Shanghai Jiao Tong University School of Medicine, Shanghai, China; ^2^ Shanghai Minimally Invasive Surgery Center, Ruijin Hospital, Shanghai Jiao Tong University School of Medicine, Shanghai, China; ^3^ Department of Traditional Chinese Medicine, Ruijin Hospital, Shanghai Jiao Tong University School of Medicine, Shanghai, China; ^4^ College of Integrated Traditional Chinese and Western Medicine, Fujian University of Traditional Chinese Medicine, Fuzhou, China

**Keywords:** Erchen decoction, colorectal cancer, network pharmacology, cell cycle, cell apoptosis

## Abstract

**Objective:** Erchen Decoction (ECD), a well-known traditional Chinese medicine, exerts metabolism-regulatory, immunoregulation, and anti-tumor effects. However, the action and pharmacological mechanism of ECD remain largely unclear. In the present study, we explored the effects and mechanisms of ECD in the treatment of CRC using network pharmacology, molecular docking, and systematic experimental validation.

**Methods:** The active components of ECD were obtained from the TCMSP database and the potential targets of them were annotated by the STRING database. The CRC-related targets were identified from different databases (OMIM, DisGeNet, GeneCards, and DrugBank). The interactive targets of ECD and CRC were screened and the protein-protein interaction (PPI) networks were constructed. Then, the hub interactive targets were calculated and visualized from the PPI network using the Cytoscape software. Gene Ontology (GO) and Kyoto Encyclopedia of Genes and Genomes (KEGG) pathway enrichment analyses were performed. In addition, the molecular docking was performed. Finally, systematic *in vitro, in vivo* and molecular biology experiments were performed to further explore the anti-tumor effects and underlying mechanisms of ECD in CRC.

**Results:** A total of 116 active components and 246 targets of ECD were predicted based on the component-target network analysis. 2406 CRC-related targets were obtained from different databases and 140 intersective targets were identified between ECD and CRC. 12 hub molecules (STAT3, JUN, MAPK3, TP53, MAPK1, RELA, FOS, ESR1, IL6, MAPK14, MYC, and CDKN1A) were finally screened from PPI network. GO and KEGG pathway enrichment analyses demonstrated that the biological discrepancy was mainly focused on the tumorigenesis-, immune-, and mechanism-related pathways. Based on the experimental validation, ECD could suppress the proliferation of CRC cells by inhibiting cell cycle and promoting cell apoptosis. In addition, ECD could inhibit tumor growth in mice. Finally, the results of molecular biology experiments suggested ECD could regulate the transcriptional levels of several hub molecules during the development of CRC, including MAPKs, PPARs, TP53, and STATs.

**Conclusion:** This study revealed the potential pharmacodynamic material basis and underlying molecular mechanisms of ECD in the treatment of CRC, providing a novel insight for us to find more effective anti-CRC drugs.

## 1 Introduction

Colorectal Cancer (CRC) is one of the most commonly diagnosed malignant tumors and is a leading cause of tumor-related mortality worldwide (Bray et al., 2018). In 2020, CRC ranked second for incidence (more than 1,900,000 cases) and third for mortality (more than 930,000 cases) globally (Sung et al., 2021). The mortality rate of CRC has decreased recently due to improvements in diagnosis and treatment (Siegel et al., 2022). Unfortunately, the prognosis of CRC patients who have been diagnosed at an advanced stage remains poor, and only a few of them are likely to benefit from the current treatments (Gbolahan and O'Neil, 2019). Moreover, many patients cannot tolerate the side effects of chemotherapy and radiotherapy, and even targeted therapy or tumor immunotherapy could bring them a considerable symptom burden (Veenstra and Krauss, 2018). Therefore, it is imperative to explore new drugs and combination therapies for CRC treatment.

In recent years, the value of Traditional Chinese medicine (TCM) in tumor treatment has been high-profile based on its low toxicity and multi-target activity (Xiang et al., 2019). Several randomized controlled trials (RCT) have been conducted to provide clinical evidence for TCM treatment of patients with different types of tumors, including CRC (Wang et al., 2021b), breast cancer (Zhang et al., 2022), lung adenocarcinoma (Li et al., 2022), and so on. Erchen Decoction (ECD), a traditional Chinese botanical formula, has been proven to play a significant role in regulating lipid metabolism and used to be applied for treating lipid-disorder-related diseases, such as obesity, hyperlipemia, and so on (Zhang et al., 2020a; Luo et al., 2020; Ding et al., 2022). Throughout the evolution of solid tumor oncology, tumor microenvironment (TME) have become increasingly recognized as a factor in tumor development and progression. To meet the demands of the TME, metabolic reprogramming occurs throughout the tumorigenesis, including lipid metabolism (Ma and Zhang, 2021), glucose metabolism (Lyssiotis and Kimmelman, 2017), ion metabolism (Andersen et al., 2014), etc. Targeting metabolism in tumors has recently been regarded as a novel therapeutic strategy for tumor therapy. Recently, Tan et al. (2020) have found that ECD could effectively inhibit the growth of laryngeal cancer (Zhou et al., 2020). CRC is also a metabolism-related malignant tumor, and the previous study indicated that ECD has a preventive effect on CRC by its regulation of tumor fatty acid metabolism. However, the actual effects of ECD on CRC and its underlying molecular functions and pathways remain poorly unknown. Thus, it will be meaningful to explore the effect and underlying mechanisms of ECD in CRC.

TCM is characterized by multiple targets and network pharmacology is a systematic method that combines molecular biology, bioinformatics, and computer science into the pharmacological study of TCM to explore their underlying mechanisms (Nogales et al., 2022). In this study, network pharmacology was firstly applied to define the effective active components of ECD and their therapeutic targets for CRC. Next, the protein-protein interaction (PPI) network analysis, molecular docking validation, and enrichment analysis were performed to predict the hub molecules of ECD for CRC treatment. Finally, a series of *in vitro* and *in vivo* experiments were utilized to verify the above molecules screened by network pharmacology. This study will explore the function of ECD for CRC treatment, understand their underlying anti-tumor mechanisms, provide evidence for the application of ECD in tumor therapy, and open new horizons for therapeutic intervention in the future.

## 2 Methods

### 2.1 Network pharmacology

#### 2.1.1 Accessing and screening the active components of erchen decoction

The keywords “*Glycyrrhiza glabra*”, “*Citrus reticulata*”, “*Pinellia ternata*” and “*Poria cocos*” were retrieved from the TCM Systems Pharmacology Database (TCMSP, https://old.tcmsp-e.com/tcmsp.php) (Ru et al., 2014). Oral bioavailability (OB) ≥ 30% and drug-likeness (DL) ≥ 0.18 were set as the threshold for screening the active components in ECD. There were 116 kinds of active components obtained from the database in total. The STRING database (https://cn.string-db.org) (Szklarczyk et al., 2021) was used to annotate targets of the active components in ECD.

#### 2.1.2 Obtaining the disease-related targets

“Colorectal Cancer” was used as the keyword to search the CRC-related targets in the online Mendelian inheritance in man (OMIM) database (Amberger et al., 2015), the database of gene disease associations (DisGeNet) (Piñero et al., 2015), GeneCards database (relevance score ≥10) (Rebhan et al., 1997) and DrugBank database (score gda ≥0.1) (Wishart et al., 2018). The CRC-related targets from different databases were merged and dereplicated. Finally, these targets were annotated by STRING.

#### 2.1.3 Analysis of protein-protein interaction network

The intersective targets were obtained between the ECD components and CRC disease targets. Then, these targets were imported into the STRING database, and the highest confidence was set as 0.9 to show the PPI network. PPI network was visualized in Cytoscape 3.9.1 (Shannon et al., 2003) software and algorithms of cytoHubba plug-in (Chin et al., 2014): Maximal Clique Centrality (MCC), Maximum Neighborhood Component (MNC), and Degree value were used to screen the top 15 hub targets from the network, respectively. Finally, intersective hub targets were obtained from three different algorithms.

#### 2.1.4 Construction of component-target network of erchen decoction

The active components in ECD and the interaction targets were constructed to the active components-targets-CRC network using Cytoscape 3.9.1 software. In addition, the top five components in the Degree value of ECD were selected to proceed molecular docking.

#### 2.1.5 Molecular docking validation

PubChem database (https://pubchem.ncbi.nlm.nih.gov/) (Kim et al., 2021) was applied to collect 2D molecular structures of active components in SDF format. Meanwhile, the 3D protein structures of hub targets in PDB format were obtained from the RCSB PDB database (https://www.rcsb.org/) (Goodsell et al., 2020). The mechanical molecular structure was optimized by Chem3D 19.0 software and transferred to PDB format as ligands. Removing water molecules from ligands and proteins, and adding hydrogen bridge to them. The docking box in proteins was calculated *via* the DeepSite database (Jiménez et al., 2017) and AutoDockTools 1.5.7 software (Goodsell and Olson, 1990) was utilized to dock ligands and proteins. Subsequently, we visualized docking results with PyMOL 2.3.4 software (Baugh et al., 2011).

#### 2.1.6 Gene ontology and kyoto encyclopedia of genes and genomes pathway enrichment analysis

The GO (Ashburner et al., 2000) and KEGG pathway enrichment analysis (Kanehisa and Goto, 2000) were performed for the 140 intersective targets using the Database for Annotation, Visualization, and Integration Discovery (David, https://david.ncifcrf.gov/) (Huang et al., 2009b) and OmicShare (Huang et al., 2009a) (https://www.omicshare.com/tools) tools. The GO enrichment analysis includes biological processes (BP), cellular component (CC), and molecular function (MF).

### 2.2 Experimental validation

#### 2.2.1 Preparation of erchen decoction

Granules of ECD were purchased from Guoyi hospital, Fujian University of TCM (Fuzhou, China), composed of *Pinellia ternata* (34.5%), *Citrus reticulata* (34.5%), *Poria cocos* (20.7%), and *Glycyrrhiza glabra* (10.3%). The ECD granules were dissolved to 0.2 g/ml in phosphate buffered saline (PBS) and filtered through a 0.22 μm pore size filter (211024-052-A, JET BIOFIL, China). Then the ECD filtrate was divided into several centrifuge tubes and stored at −20°C for future use.

#### 2.2.2 Cell lines and cell culture

The human colorectal cancer cell lines HT29, SW620, and DLD-1, were obtained from the American Type Culture Collection (ATCC). Consistent with the previous methods (Shao et al., 2021), all cells were stored at the Shanghai Institute of Digestive Surgery and cultured in the RPMI 1640 medium (meilunbio, Dalian, China) with 10% Fetal Bovine Serum (FBS, Gibco, NY, United States) at 37°C with 5% CO_2_.

#### 2.2.3 Cell viability assay

The working concentrations of ECD were diluted in complete medium to 0, 0.3125, 0.625, 1.25, 2.5, 5, 10, 20, 40, and 80 mg/ml. Three wells (repeats) were applied for each concentration and repeated at least 3 times. After the gradient concentrations of ECD treatments for 24 h, 48 h, or 72 h, cell viability assays were measured by Cell Counting Kit 8 (CCK8, meilunbio, Dalian, China). Then, the half-maximal inhibitory concentration (IC_50_) values of each cell line based on different concentrations and times of ECD treatment were determined using nonlinear regression. Finally, the cell viability of each cell line with low (IC_25_), medium (IC_50_), and high concentrations (IC_75_) of ECD treatment was also further tested in different periods.

#### 2.2.4 Colony formation assay

Three CRC cell lines were all plated on 6-well plates at 1,500 cells per well and treated with different concentrations of ECD for 12 h. Then all cells continued to be cultured in a complete medium for 2 weeks. Colonies were fixed by 4% paraformaldehyde for 30 min and then stained with 1% crystalline violet solution for 20 min. The colonies were observed by the light microscope and manually counted in a blinded fashion to measure the cell colony numbers (at least three random fields).

#### 2.2.5 5-Ethynyl-2′-deoxyuridine incorporation assay

Three CRC cell lines were all plated on 6-well plates at 2 × 10^4^ cells per well and cultured for 24 h. Cells were treated with the low (IC_25_), medium (IC_50_), and high concentrations (IC_75_) of ECD for 48 h. Then, 10 μM EdU (Cellorlab, Shanghai, China) was added and incubated for 2 h. The remaining operations, including fixing, glycine incubating, and dilution, were performed based on the standard protocols in a light-repelled environment. The fluorescence images of these cells were captured under an inverted fluorescence microscope (Nikon, Tokyo, Japan) and at least three random fields in each well were photographed.

#### 2.2.6 Flow cytometry assay for the cell cycle and cell apoptosis

Three CRC cell lines were all plated and cultured on 6-well plates for 24 h and treated with the low (IC_25_), medium (IC_50_), and high concentrations (IC_75_) of ECD for 48 h. For the cell cycle assay, the cells (at least 1 × 10^6^) were fixed by 75% ethanol and stored at 4°C overnight. After washing twice with cold PBS, 300 μl PI/RNase staining (#550825, BD Biosciences, United States) was added to each sample and incubated for 30 min at 37°C under protection from light. The cell cycle was then analyzed by flow cytometry (FACSCalibur; Becton Dickinson, United States). Similarly, for the cell apoptosis assay, both floating and attached cells in each well were harvested and washed twice with cold PBS. Then, cells were incubated with 3 μl FITC-Annexin V (#556419, BD Biosciences, United States) and 5 μl PI (#556463, BD Biosciences, United States) in 100 μl binding buffer at 37°C under protection from light. Next, 200 μl binding buffer was added to each sample, and cell apoptosis was also measured by flow cytometry.

#### 2.2.7 RNA Isolation and Real-Time PCR

RNA Isolation and Real-Time PCR were consistent with previous methods (Shao et al., 2021). The complete information of each RNA primer used in this study was listed in Supplementary Table S1. The relative mRNA expression levels of them were calculated by the 2^−ΔΔCt^ method and normalized against that of *β-Actin*.

#### 2.2.8 Western bolt

Western blot was also performed as in previous methods (Shao et al., 2021). Primary antibodies: β-Tubulin (A12289, ABclonal, China), p38 MAPK (A4771, ABclonal, China), and p21 (ab109199, Abcam, Britain). Secondary antibody: anti-rabbit (30000-0-AP, Proteintech, United States).

#### 2.2.9 *In Vivo* anti-tumor assay and immunohistochemistry

Nude mice (BALB/cA-nu mice, 6 weeks old, 20 ± 2 g) were purchased from Shanghai Jihui Laboratory Animal Care Co., Ltd. (China). All animal experiments in this study were conducted based on the Community guidelines of the animal care and use and were approved by the Ethics Committee of Fujian University of Traditional Chinese Medicine (No. 3501020096434). For the construction of the subcutaneous xenograft mouse models, 3 × 10^6^ HT29 cells were subcutaneously injected into the middle and rear armpit of individual nude mice (at 0.1 ml each). Seven days after cell injection, mice were randomly divided into four groups: control (*n* = 5, normal saline, 0.2 ml/20 g), ECD-L, ECD-M, and ECD-H (*n* = 5, ECD at 200, 400, and 600 mg/kg, 0.2 ml/20 g, respectively). ECD was dissolved in a PBS solution and delivered daily by intragastric administration for 14 days. Then, tumor volumes were measured every 3 days and calculated by the formula: length × width^2^/2. At the end of the experiment, mice were sacrificed and tumors were photographed, weighed, and stored. H and E staining and Immunohistochemistry (IHC) of the paraffin-embedded tumor tissue sections were performed based on the standard protocols. Antibodies for IHC: p21 (A19094, ABclonal, China) and p38 MAPK (A10832, ABclonal, China).

### 2.3 Statistical analysis

All the statistical analysis was performed by the R (version: 3.4.0) and SPSS (version: 25) software for double checking in this study. All experiments were repeated more than three times, and the corresponding results were presented as means ± SD. Student t and one-way ANOVA tests were used to compare two or more groups. Categorical variables were analyzed by chi-square test. *p* < 0.05 was regarded as a statistically significant difference.

## 3 Results

### 3.1 Main active components and targets of erchen decoction

**FIGURE 1:**
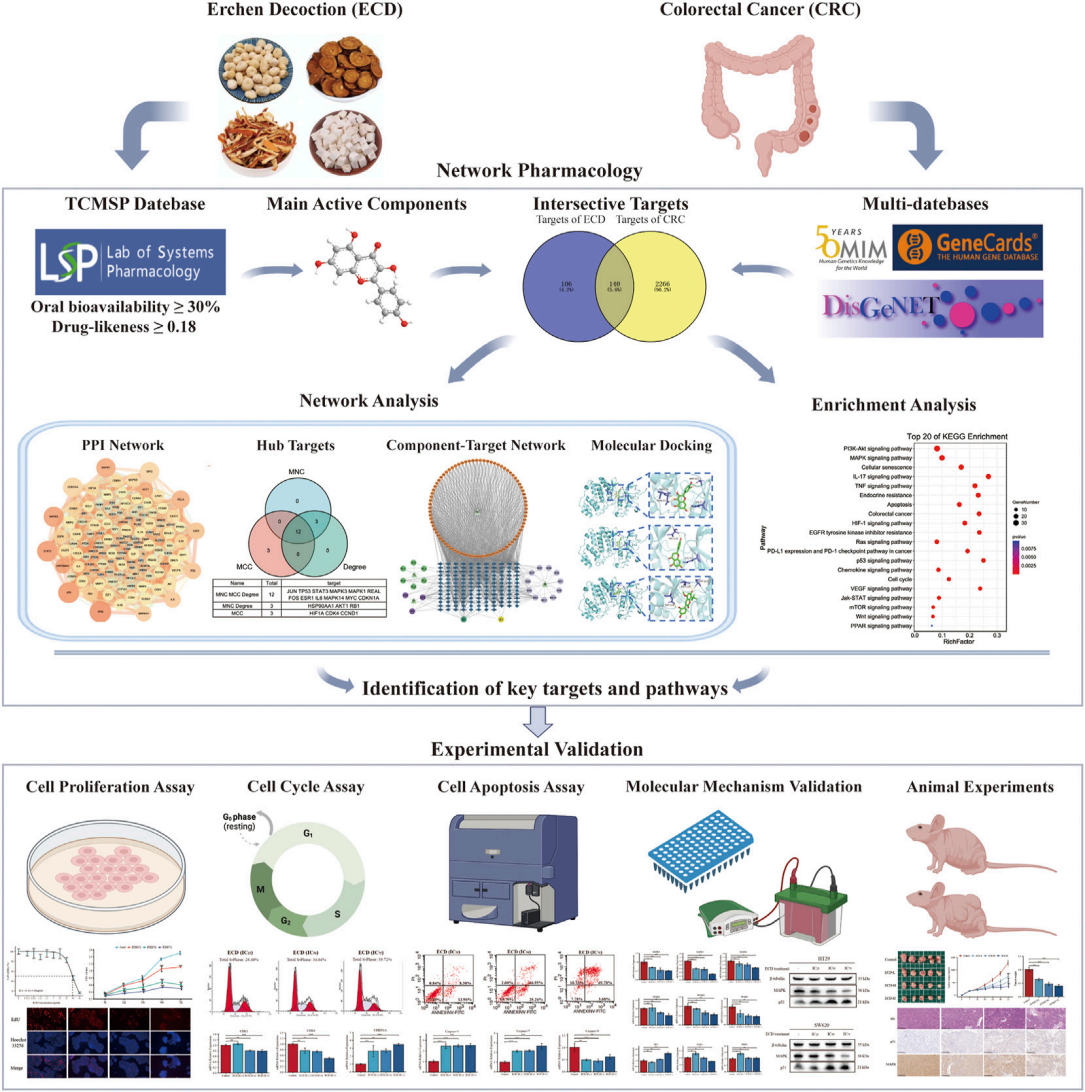
Schematic flowchart of the study design.

A schematic flow chart of the study was shown in Figure 1. 116 components of ECD and 246 corresponding underlying targets were screened by OB and DL values from the TCMSP database. The information of the top two active components with the OB value of each herb in ECD was shown in Table 1.

**TABLE 1:** Top 2 active components with OB value of each herb in ECD.

MOL ID	Herbs	Active components	OB%	DL
MOL006957	*Pinellia ternata*	(3S,6S)-3-(benzyl)-6-(4-hydroxybenzyl)piperazine-2,5-quinone	46.89	0.27
MOL006967	*Pinellia ternata*	Beta-D-Ribofuranoside	44.72	0.21
MOL005815	*Citrus reticulata*	Citromitin	86.9	0.51
MOL005828	*Citrus reticulata*	Nobiletin	61.67	0.52
MOL000282	*Poria cocos*	Ergosta-7,22E-dien-3beta-ol	43.51	0.72
MOL000283	*Poria cocos*	Ergosterol peroxide	40.36	0.81
MOL002311	*Glycyrrhiza glabra*	Glycyrol	90.78	0.67
MOL004990	*Glycyrrhiza glabra*	7,2′,4′-trihydroxy-5-methoxy-3-arylcoumarin	83.71	0.27

### 3.2 Intersective targets between erchen decoction and colorectal cancer

2406 CRC-related targets were obtained from four different databases, including OMIM, DisGeNet, GeneCards, and DrugBank. Besides, these targets from different databases were screened and deduplicated. A Venn diagram (Supplementary Figure S1) was drawn to show the 140 intersective targets between ECD and CRC.

### 3.3 Analysis of protein-protein interaction network and screening hub molecules

**FIGURE 2:**
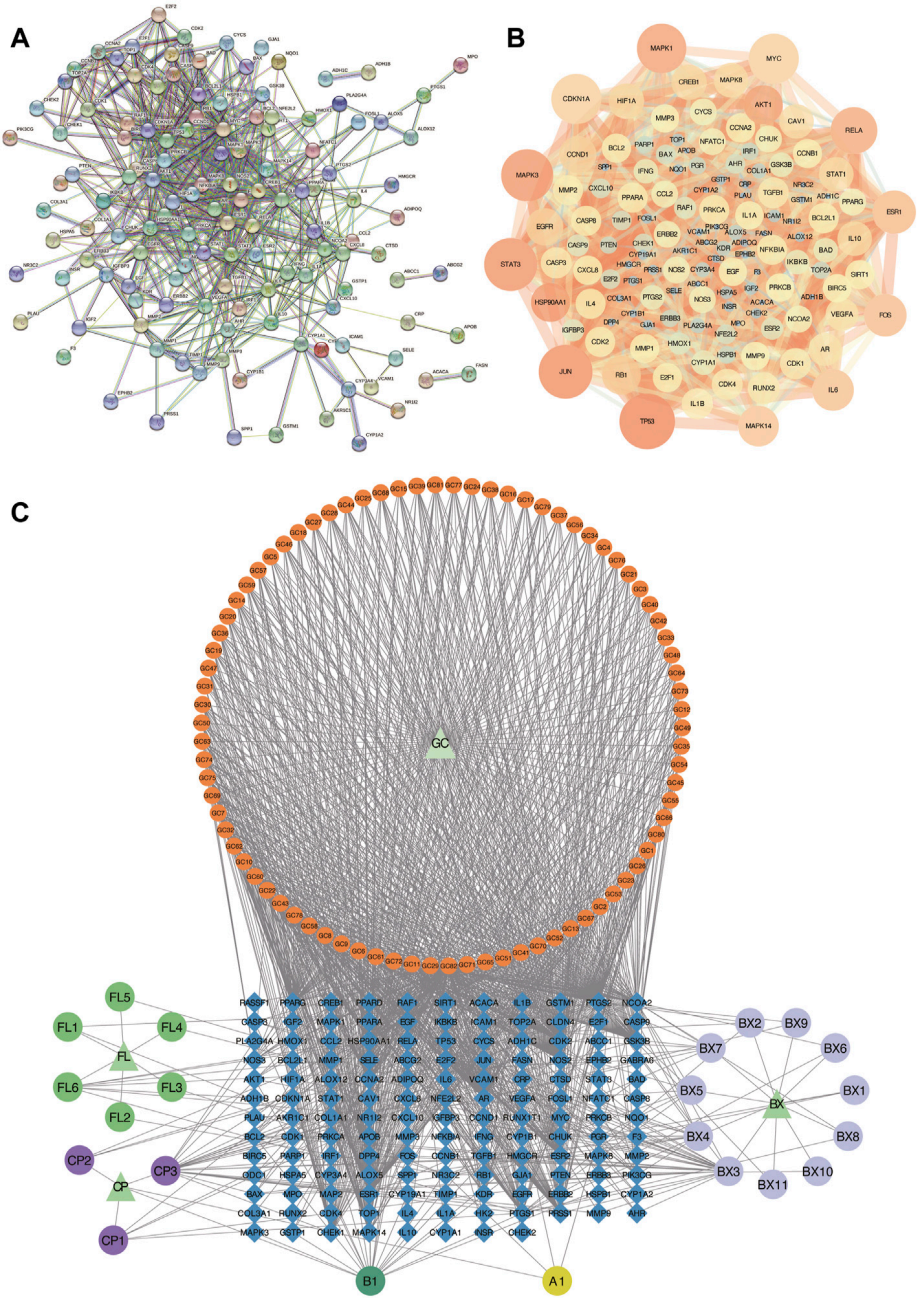
Analysis of PPI and Component-Target-Disease networks. **(A,B)** PPI Networks of the 140 intersective targets. **(C)** The component-Target-Disease network of herb components and CRC-related targets. GC: *Glycyrrhiza glabra*; BX: *Pinellia ternata*; FL: *Poria cocos*; CP: *Citrus reticulata*.

**FIGURE 3:**
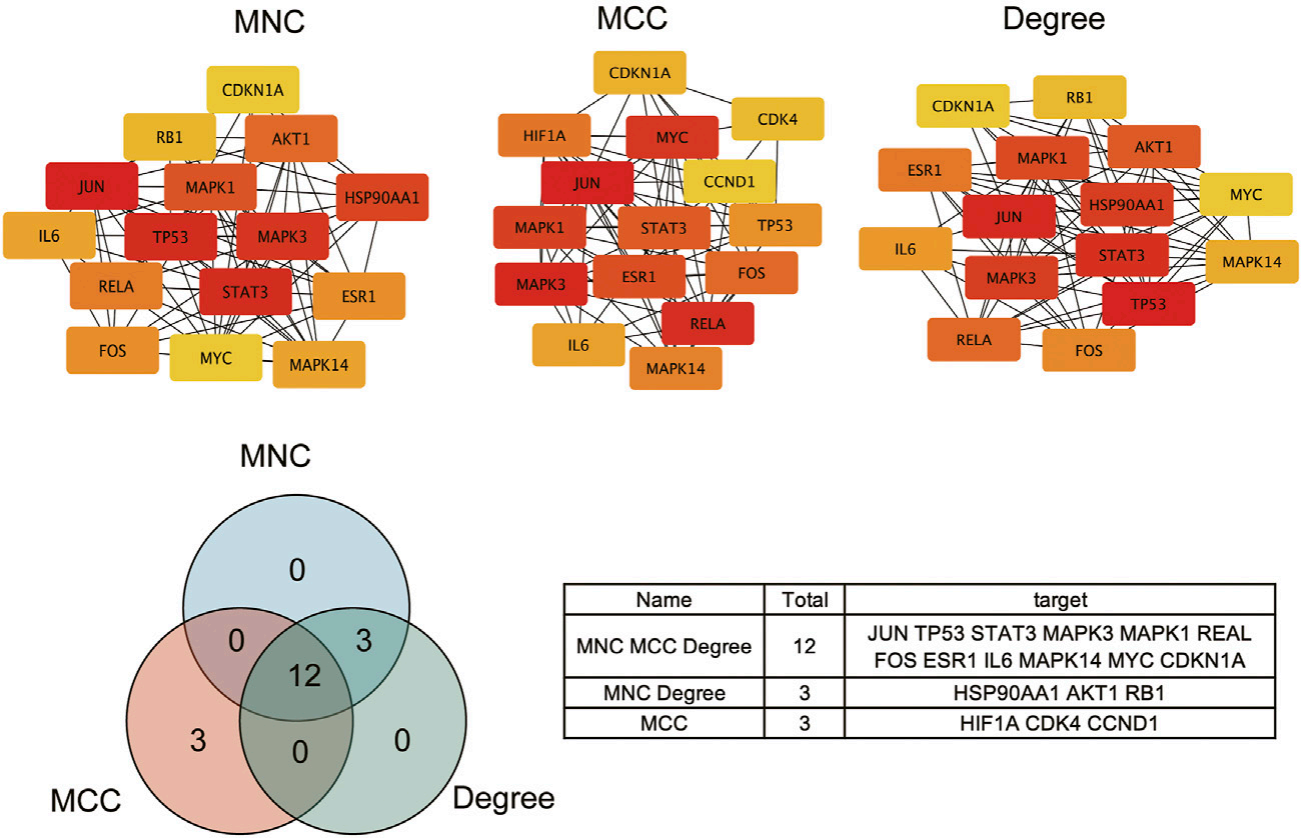
Screening intersective hub targets.

To further explore the relationship among the 140 intersective target molecules, the PPI network was constructed by the STRING database, containing 132 nodes and 690 edges, as shown in Figures 2A,B. Besides, the size and color depth of the nodes and edges were regulated to represent the connectivity of nodes. Larger size and darker color, indicate the stronger connection between the targets in the network. The Component-Target-Disease network (Figure 2C) was drawn to demonstrate the relationship between herb components and CRC-related targets, including 248 nodes and 1,248 edges. And the full names of the abbreviations in Figure 2C were shown in Supplementary Table S2. The greater area of the graph indicated the higher Degree value (the connectivity between nodes). 12 hub targets were finally screened from the PPI network *via* four algorithms and shown in Figure 3, including STAT3, JUN, MAPK3, TP53, MAPK1, RELA, FOS, ESR1, IL6, MAPK14, MYC, and CDKN1A.

### 3.4 Validation of molecular docking

**FIGURE 4:**
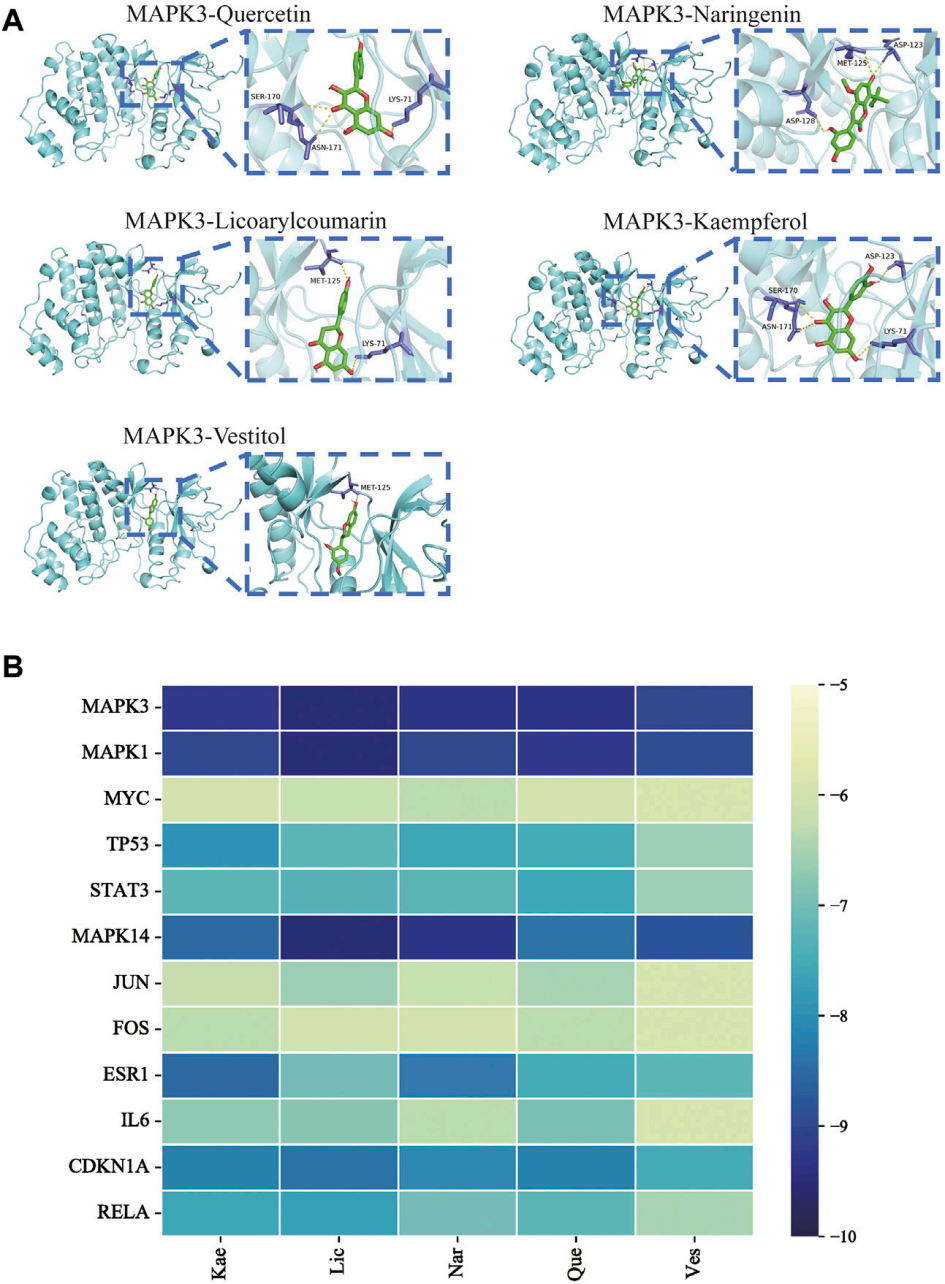
Validation of molecular docking. **(A)** Representative molecular docking mode diagram. **(B)** Heatmap of binding between key active components and intersective hub targets.

To verify the correlation between ECD and CRC, molecular docking, a novel method, was performed by PyMOL software. The top five main active components were screened by Degree value, including Quercetin (Que), Naringenin (Nar), Licoarylcoumarin (Lic), Kaempferol (Kae), and Vestitol (Ves), displayed as 2D structure in Table 2. Then, five active components and 12 hub targets were combined as ligands and receptors, respectively. Therefore, the lowest binding energy from the docking between ligands and receptors were screened as shown in Figure 4A. The heatmap plot was drawn to show all the docking degrees between them in Figure 4B; Table 3.

**TABLE 2:** 2D structure of top 5 active components screened by degree value.

MOL ID	Molecule name	Structure	Degree	OB%	DL
MOL000098	Quercetin	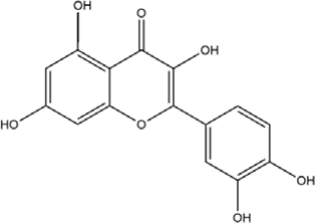	109	46.43	0.28
MOL004328	Naringenin	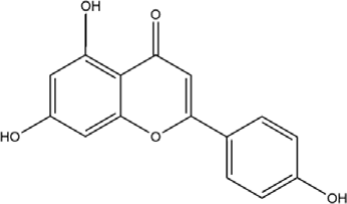	46	59.29	0.21
MOL004849	Licoarylcoumarin	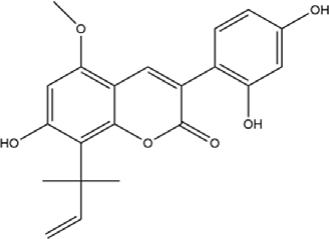	38	59.62	0.43
MOL000422	Kaempferol	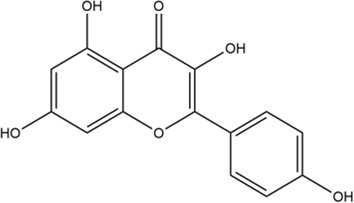	36	41.88	0.24
MOL000500	Vestitol	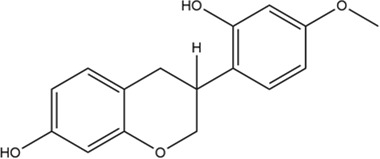	31	74.66	0.21

**TABLE 3:** Binding energy of main active components in ECD with hub targets.

Hub targets	Binding energy (kcal/mol)				
	Kaempferol	Licoarylcoumarin	Naringenin	Quercetin	Vestitol
MAPK3	−9.2	−9.5	−9.3	−9.3	−9.0
MAPK1	−9.0	−9.5	−9.0	−9.2	−8.9
MYC	−6.0	−6.1	−6.3	−6.0	−5.9
TP53	−7.9	−7.2	−7.6	−7.5	−6.6
STAT3	−7.2	−7.3	−7.2	−7.6	−6.6
MAPK14	−8.5	−9.5	−9.3	−8.4	−8.8
JUN	−6.2	−6.6	−6.1	−6.5	−5.9
FOS	−6.3	−6.0	−6.0	−6.3	−5.9
ESR1	−8.5	−7.0	−8.3	−7.5	−7.2
IL6	−6.7	−6.8	−6.3	−6.9	−5.9
CDKN1A	−8.2	−8.4	−8.1	−8.2	−7.5
RELA	−7.6	−7.7	−7.0	−7.2	−6.5

### 3.5 Analysis of functional enrichment

**FIGURE 5:**
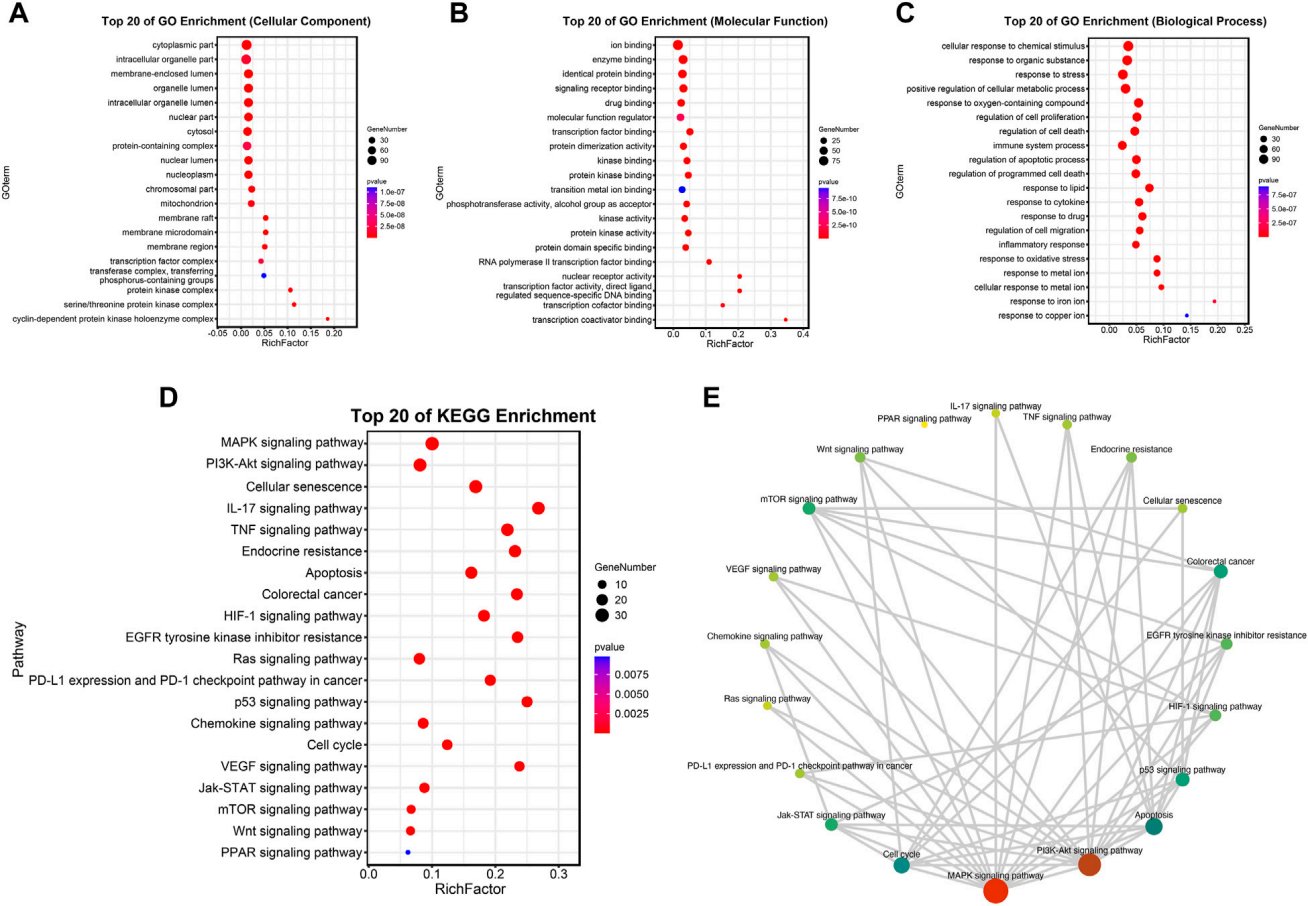
Analysis of functional enrichment. **(A–C)** Top 20 cellular components **(A)**, top 20 molecular functions **(B)** and top 20 biological processes **(C)** in the GO enrichment analysis. **(D,E)** Top 20 pathways in the KEGG enrichment analysis.

To further explore the above molecules of the PPI network, a bubble diagram was drawn to show the results of GO and KEGG enrichment analysis concisely. The top 20 enrichment processes of GO enrichment analysis were shown in Figures 5A–C. These items are primarily centralized in the regulation of cell proliferation and apoptotic, intracellular organelle part, molecular function regulator, etc. Consistently, the biological discrepancy was also focused on the tumorigenesis-, immune-, and mechanism-related pathways, such as MAPK signaling pathway, PI3K/Akt signaling pathway, Ras signaling pathway, p53 signaling pathway, and cell-cycle-associated pathways; IL-17 signaling pathway, TNF signaling pathway, and chemokine signaling pathway; Endocrine resistance by KEGG pathway enrichment analysis in Figures 5D,E.

### 3.6 Erchen decoction inhibits the proliferation of colorectal cancer cells

**FIGURE 6:**
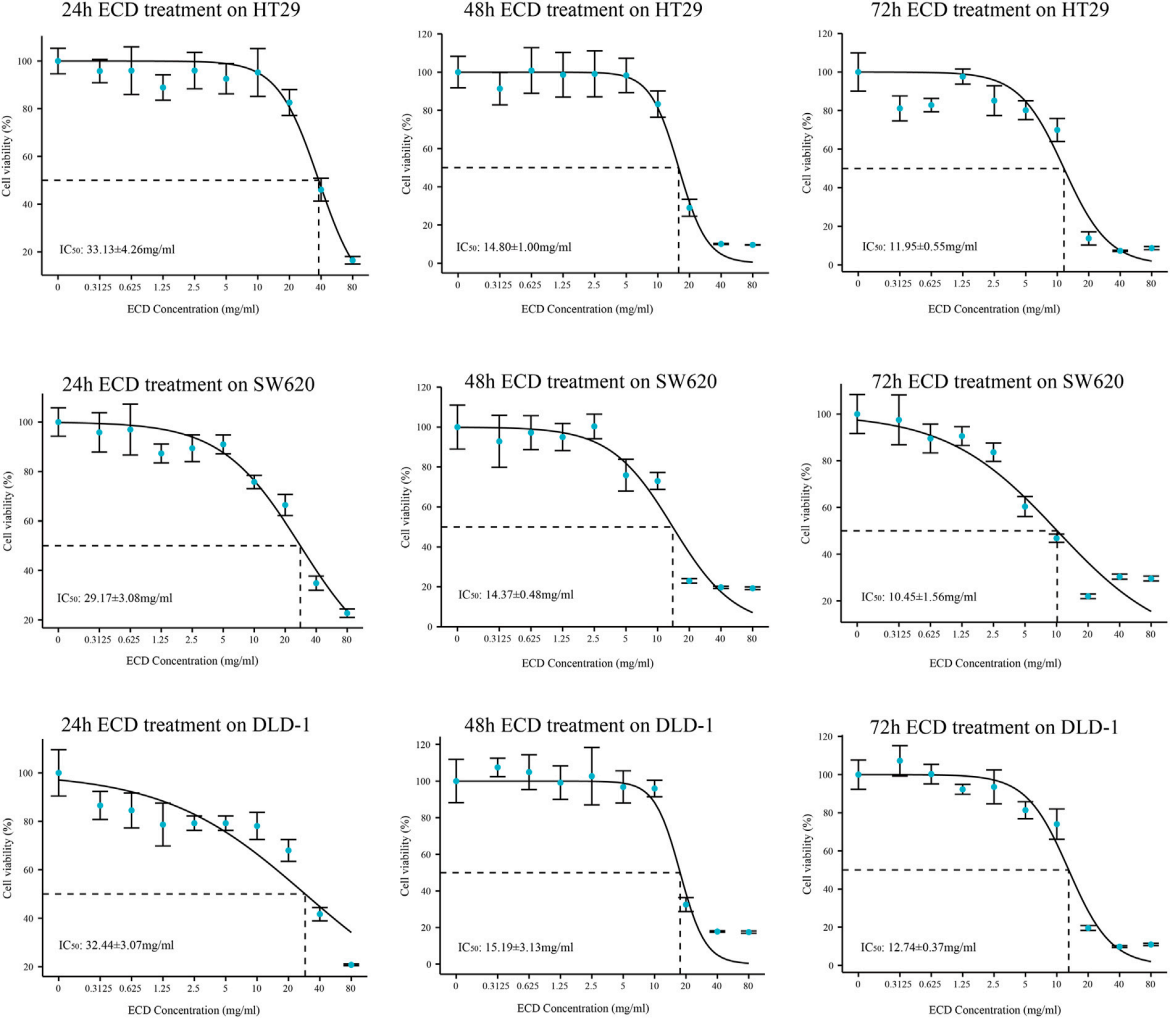
The IC_50_ values of ECD in each CRC cell line (HT29, SW620, and DLD-1).

**FIGURE 7:**
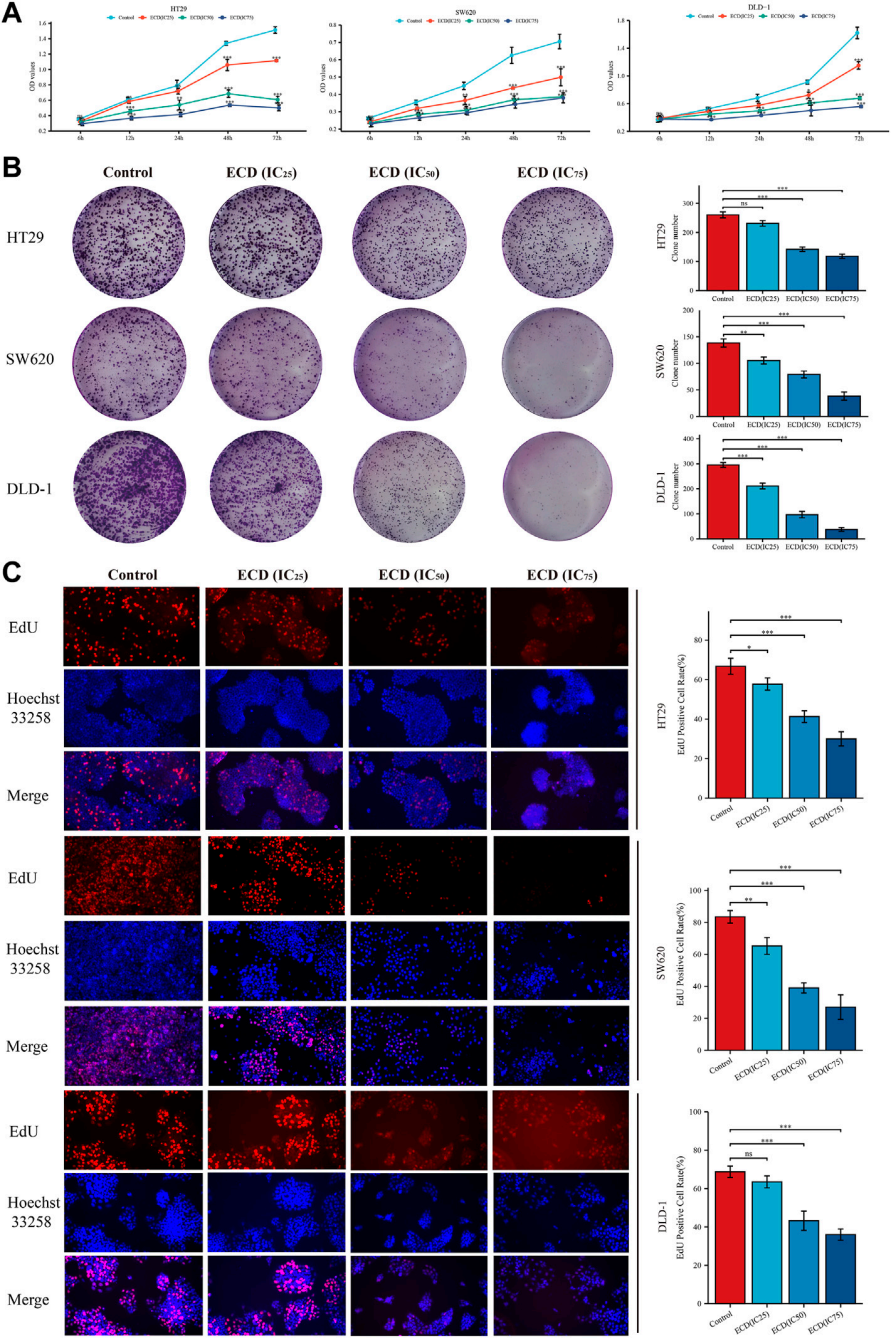
ECD inhibited the proliferation of CRC cell lines. **(A)** Cell Counting Kit-8 (CCK-8) analyses of HT29, SW620, and DLD-1 cell lines after treatment with different working concentrations (IC_25_, IC_50_, and IC_75_) of ECD for 6, 12, 24, 48, and 72 h (*n* = 3 per group). **(B)** Colony formation analyses of HT29, SW620, and DLD-1 cell lines cultured with different working concentrations (IC_25_, IC_50_, and IC_75_) of ECD for 48 h (*n* = 3 per group). **(C)** EdU assays of HT29, SW620, and DLD-1 cell lines treated with different working concentrations (IC_25_, IC_50_, and IC_75_) of ECD for 48 h (× 200) (*n* = 3 per group). ^ns^ *P* > 0.05, ^*^ *p* < 0.05, ^**^ *p* < 0.01, ^***^ *p* < 0.001.

**FIGURE 8:**
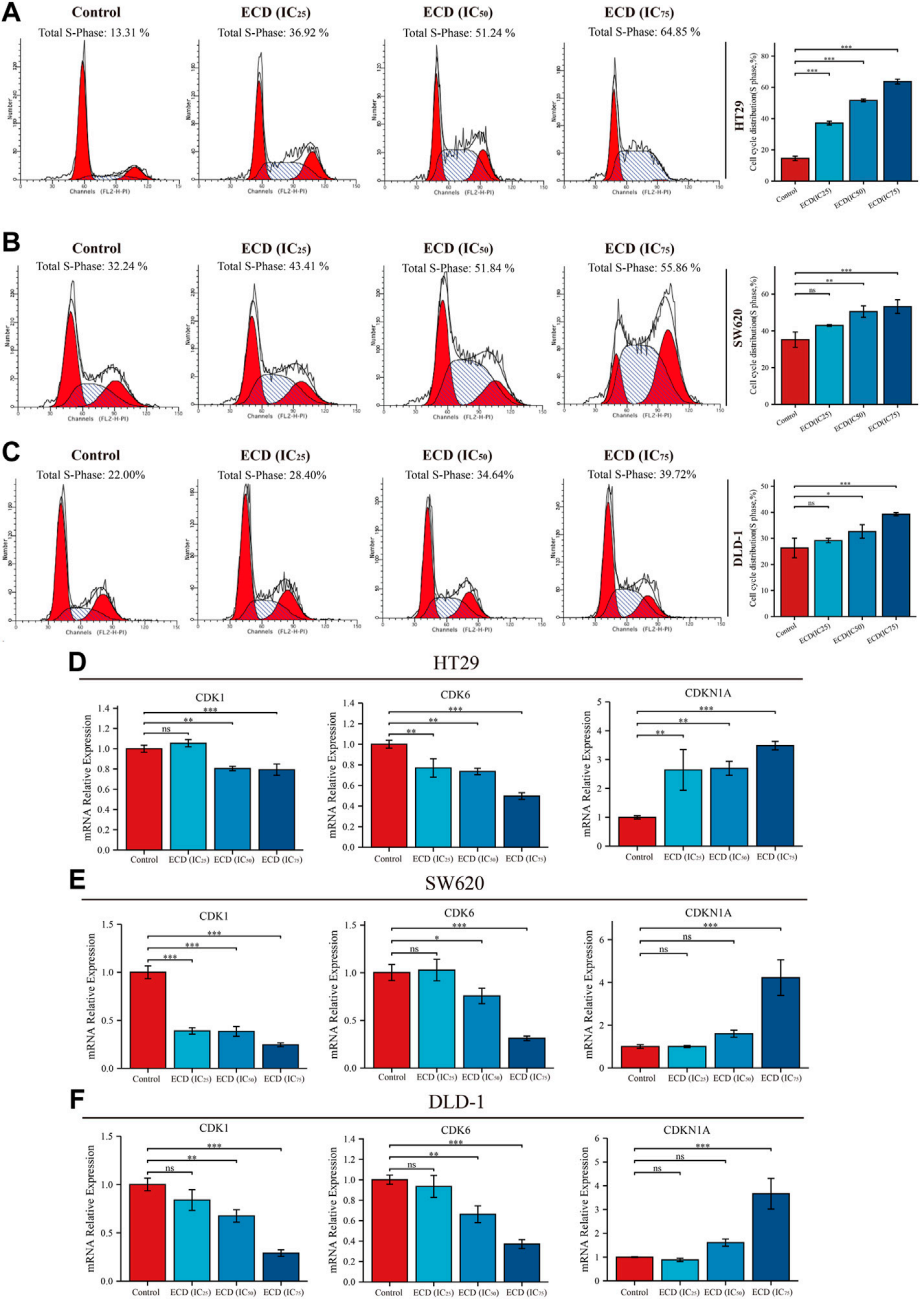
ECD inhibited cell cycle of CRC cells. A-C. Cell cycle analyses of HT29 **(A)**, SW620 **(B)**, and DLD-1 **(C)** cell lines treated with different working concentrations (IC_25_, IC_50_, and IC_75_) of ECD for 48 h measured by flow cytometry assay (*n* = 3 per group). D-F. The mRNA relative expression of CDK1, CDK6, and CDKN1A in HT29 **(D)**, SW620 **(E)**, and DLD-1 **(F)** cell lines treated with different working concentrations (IC_25_, IC_50_, and IC_75_) of ECD for 48 h measured by real-time PCR (*n* = 3 per group). ^ns^ *P* > 0.05, ^*^ *p* < 0.05, ^**^ *p* < 0.01, ^***^ *p* < 0.001.

In order to explore the inhibitory effect of ECD in CRC, CRC cell lines (HT29, SW620, and DLD-1) were selected and treated with different working concentrations of ECD (0–80 mg/ml) for 24, 48, and 72 h respectively. As shown in Figure 6, the results of the cell viability assays and the IC_50_ values of each cell line suggest that ECD could significantly inhibit the proliferation of CRC cells in a time- and dose-dependent manner. Considering the time-preference effects of ECD, the compromised duration (48 h) has been chosen to conduct the follow-up experiments. In this study, the IC_50_ values for HT29, SW620, and DLD-1 cell lines were 14.80 ± 1.00 mg/ml, 14.37 ± 0.48 mg/ml and 15.19 ± 3.13 for 48 h. CCK-8 assay, colony formation assay, and EdU assays were utilized to investigate the effects of ECD on the proliferation of CRC cell lines. CCK-8 assays further showed the cytotoxicity of ECD in manners of dose- and time-dependence (Figure 7A). ECD could inhibit the colony formation of each CRC cell line (Figure 7B) and reduce the percentage of EdU-positive cells (Figure 7C) in a dose-dependent manner. To further explore the inhibitory mechanisms of ECD in CRC, cell cycle analysis indicated that the percentage of each cell in the S phase increased significantly with the increasing concentrations of ECD, while that of the G0/G1 phase decreased significantly compared with corresponding controls (Figures 8A–C). The mRNA expression levels of *CDK1* and *CDK6* were down-regulated at the indicated concentrations of ECD, while those of *CDKN1A* was up-regulated on all CRC cell lines (Figures 8D–F). These results clearly showed that ECD has anti-proliferative effects on CRC cells by affecting the transcriptional levels of several cell-cycle-related molecules.

### 3.7 Erchen decoction induced apoptosis of colorectal cancer cells

**FIGURE 9:**
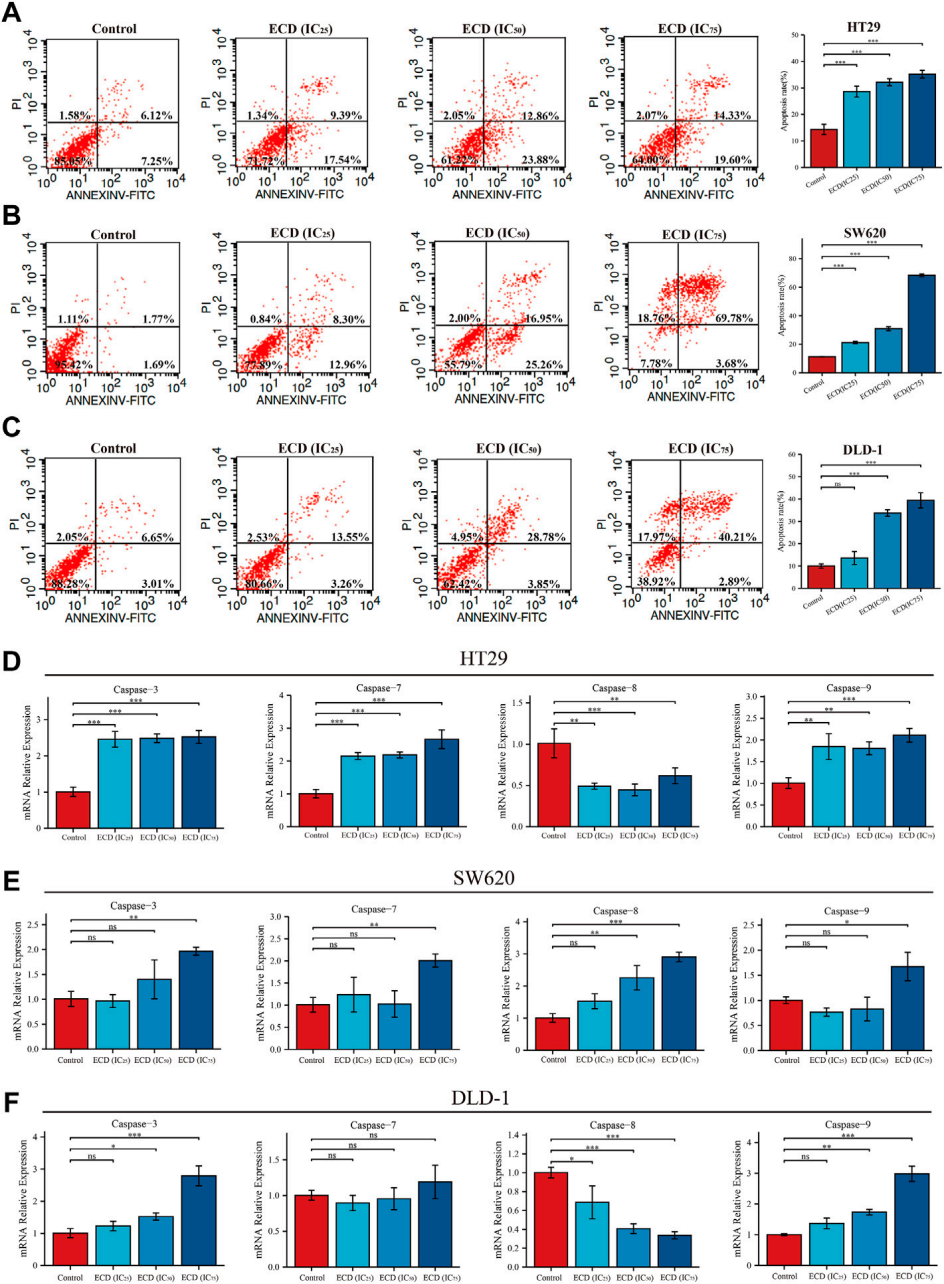
ECD induced cell apoptosis of CRC cells. **(A–C)** Cell apoptosis analyses of HT29 **(A)**, SW620 **(B)**, and DLD-1 **(C)** cell lines treated with different working concentrations (IC_25_, IC_50_, and IC_75_) of ECD for 48 h measured by flow cytometry assay (*n* = 3 per group). D-F. The mRNA relative expression of CASP3, CASP7, CASP8, and CASP9 in HT29 **(D)**, SW620 **(E)**, and DLD-1 **(F)** cell lines treated with different working concentrations (IC_25_, IC_50_, and IC_75_) of ECD for 48 h measured by real-time PCR (*n* = 3 per group). ^ns^ *P* > 0.05, ^*^ *p* < 0.05, ^**^ *p* < 0.01, ^***^ *p* < 0.001.

With the increasing concentrations of ECD, the significant reductions in the number of CRC cell lines, abnormal cell morphology (wrinkling, shrinkage or nuclear vacuolization, etc.), and increasing floating cells and cell debris were observed from the light microscopy images (Supplementary Figure S1). These findings suggest that ECD has cytotoxic effects and may induce the apoptosis of CRC cell lines. Therefore, the flow cytometry assays were applied to further detect the pro-apoptotic effects of ECD in CRC. The results indicated that the total apoptosis rates of each CRC cell line increased in a dose-dependent manner (Figures 9A–C). In addition, ECD promoted the mRNA expression levels of the caspase family, including the initiator caspase nine and the effector caspases (caspases 3 and 7), as shown in Figures 9D–F.

### 3.8 Underlying molecular mechanisms of erchen decoction in colorectal cancer treatments

**FIGURE 10:**
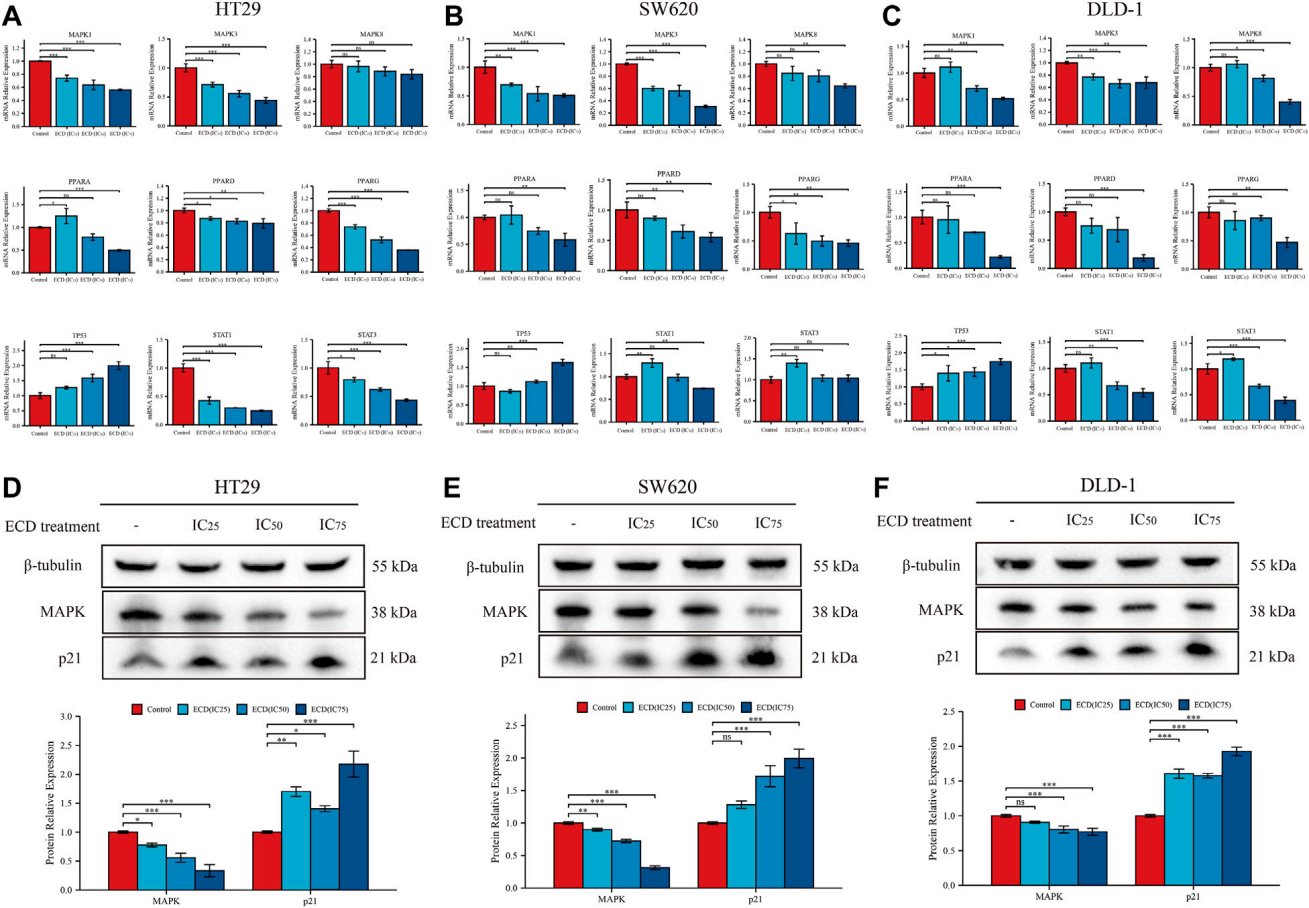
Underlying molecular mechanisms of ECD in CRC treatments. **(A–C)** The mRNA relative expression levels of MAPKs, PPARs, STATs, and TP53 in HT29 **(A)**, SW620 **(B)**, and DLD-1 **(C)** cell lines treated with different working concentrations (IC_25_, IC_50_, and IC_75_) of ECD for 48 h measured by real-time PCR (*n* = 3 per group). D-F. The protein relative expression of MAPK and p21 in HT29 **(D)**, SW620 **(E)**, and DLD-1 **(F)** cell lines treated with different working concentrations (IC_25_, IC_50_, and IC_75_) of ECD for 48 h measured by western blot (*n* = 3 per group). ^ns^ *P* > 0.05, ^*^ *p* < 0.05, ^**^ *p* < 0.01, ^***^ *p* < 0.001.

According to the results of network pharmacology, several key molecules (MAPK1, MAPK3, MAPK14, TP53, STAT3, CDKN1A, JUN, RELA, FOS, ESR1, IL6, and MYC) and pathways (MAPK signaling pathway, p53 signaling pathway, Apoptosis, Cell cycle and so on) were predicted to be underlying molecular mechanisms of ECD in CRC treatments. Surprisingly, the results of RT-PCR showed that ECD could regulate the transcriptional levels of MAPKs (MAPK1, MAPK3, MAPK8), PPARs (PPARA, PPARD, PPARG), TP53, and STATs (STAT1 and STAT3) in each CRC cell line (Figures 10A–C). After treating with different working concentrations of ECD, the mRNA expression levels of MAPKs, PPARs, and STATs in the CRC cell lines significantly decreased, while those of TP53 increased compared to the respective control groups. In addition, ECD downregulated the translational levels of MAPK and upregulated the translational levels of the cycle-related protein (p21) in CRC cell lines (Figures 10D–F). In conclusion, these findings suggest that ECD exerts anti-tumor effects by regulating the transcription and translation of several hub molecules in the development and progression of CRC.

### 3.9 Erchen decoction inhibited tumor growth in mice

**FIGURE 11:**
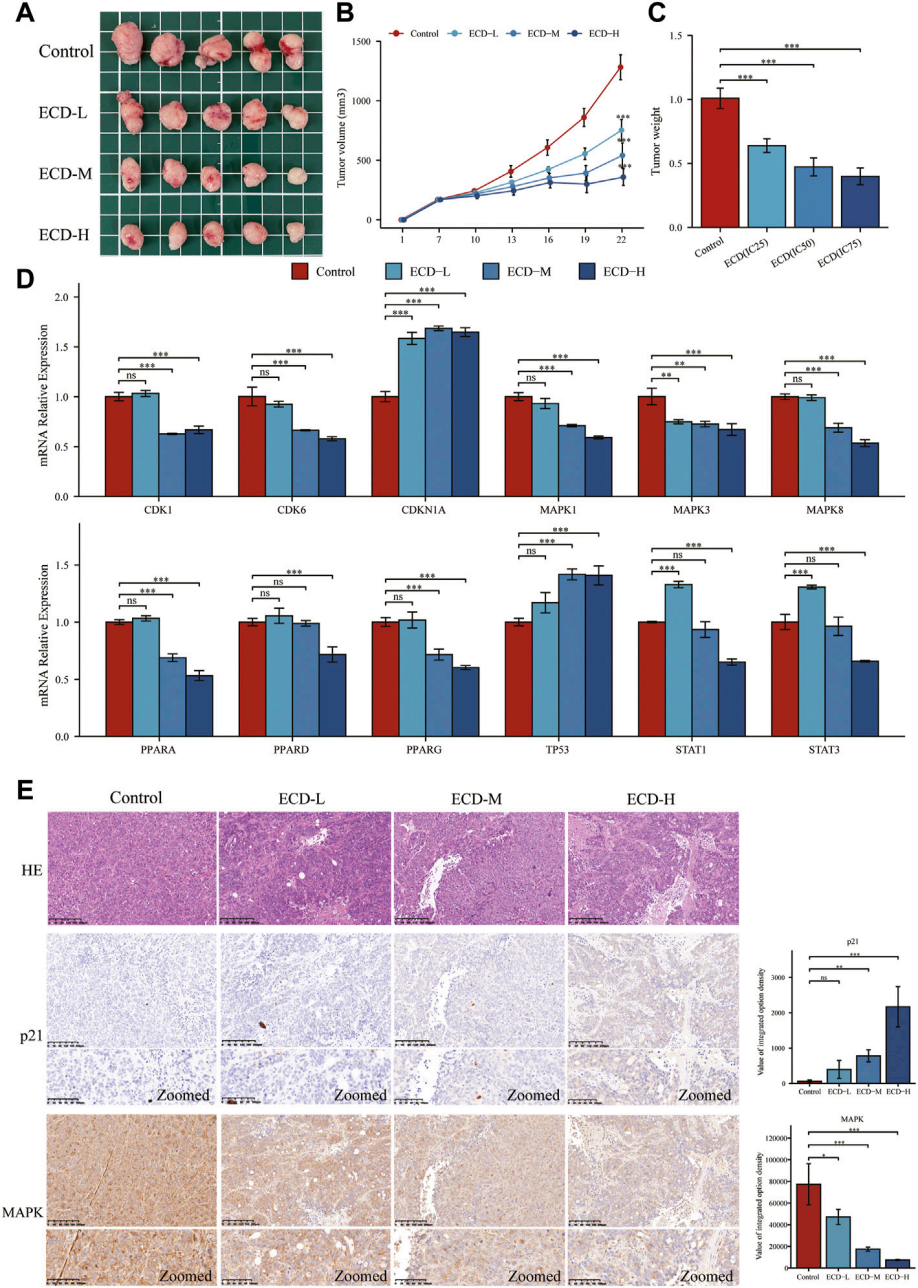
ECD inhibited tumor growth in mice. **(A)** The anti-tumor effects of ECD in mice. **(B)** Variations in tumor volume in mice during ECD treatment (*n* = 5). **(C)** Variations in tumor weights after ECD treatment (*n* = 5). **(D)** The mRNA relative expression levels of CDKs, CDKN1A, MAPKs, PPARs, STATs, and TP53 in tumor xenografts after ECD treatment. **(E)** HE staining and IHC results of tumor xenografts in different groups. ^ns^ *P* > 0.05, ^*^ *p* < 0.05, ^**^ *p* < 0.01, ^***^ *p* < 0.001.

To further explore the anti-tumor effects of ECD *in vivo*, the HT29 cell xenograft tumor models were established. The results showed that ECD could significantly inhibit tumor growth in a dose-dependent manner (Figure 11A). The tumor volume and tumor weight in ECD-L, ECD-M, and ECD-H groups were markedly reduced compared to those in the control group (Figures 11B,C). Consistent with the *in vitro* results, the *in vivo* results demonstrated that ECD could also regulate the transcriptional levels of several cell-cycle-related molecules, MAPKs, PPARs, TP53, and STATs in HT29 xenografts (Figure 11D). In addition, the results of H&E staining showed that there were obvious necrosis in the tumor tissues after ECD intervention and IHC results also confirmed that ECD significantly upregulated the protein levels of p21 and downregulated the protein levels of MAPK in HT29 xenografts (Figure 11E).

## 4 Discussion

The traditional Chinese herbal medicine formulas have low toxicity and have multi-target activity for preventing or treating various diseases, including cardiovascular disease (Zhang et al., 2020b), endocrine disease (Zhang et al., 2020a), hematologic disease (Chiu et al., 2021) and cancers (Lu et al., 2021), etc. The occurrence and development of CRC is thought to be the results of a combination of multiples genes, factors, stages (Li et al., 2015), which indicates that the multi-component and multi-target activity of Chinese medicine seems to be more beneficial to the treatment of CRC individuals (Wang et al., 2021b; Sun et al., 2021). With the advancement in the understanding of tumor biology and the microenvironment, cancer metabolic reprogramming has received a great deal of attention and is now recognized as a major avenue for the treatment of tumors (Pavlova and Thompson, 2016; Xia et al., 2021). ECD is the common TCM that originated from the Taiping Huimin Formula Bureau and has been widely applied in the treatment of obesity, hyperlipidemia, diabetes, and other diseases caused by metabolic disorders (Gao et al., 2015; Ding et al., 2018; Zhang et al., 2020a; Zhao et al., 2021). Considering the remarkable metabolic regulation effects of ECD, recent studies indicated that ECD could inhibit tumorigenesis and promote the apoptosis of laryngeal squamous cell carcinoma cells (Tan et al., 2020; Zhou et al., 2020). While some clinical studies showed ECD could be utilized as adjuvant therapy for patients with advanced CRC (Mei et al., 2016; Zhou et al., 2021), the anti-tumor effects and mechanisms of ECD on CRC have not been systematically determined. Therefore, it could be meaningful to investigate the effects and mechanisms of ECD in the treatment of CRC.

However, the basic studies and widespread clinical applications of Chinese medicine still face great challenges because of the complexity and diversity of the chemical components. And it is also difficult for us to ensure the active components and the pharmacodynamic mechanisms of ECD after entering the body. Fortunately, with the development of bioinformatics technology and the construction of a TCM database, network pharmacology is a new insight for studying TCM (Hopkins, 2008). The theoretical concepts of network pharmacology are similar to the multi-components and multi-targets of TCM, and it is an effective and clear method of analyzing drugs, components, and diseases targets (Li, 2016). Therefore, to further explore the pharmacodynamic effects and mechanisms of ECD for treating CRC, the network pharmacology analysis, molecular docking, GO and KEGG pathway enrichment analysis, and systematic experimental validation were performed in the current study.

Firstly, among 116 screened components of ECD, Kaempferol, Naringenin, Licoarylcoumarin, Quercetin, and Vestitol were identified as core active components. Kaempferol has been shown to inhibit several cancers by regulating the reactive oxygen species (ROS) in TME (Imran et al., 2019; Wang et al., 2021a). Studies have shown that Naringenin could provide cancer prevention and therapy through the modulation of TME and has been considered as a potential immunomodulator in tumor therapeutics (Casey et al., 2015; Zeng et al., 2018). Quercetin has been reported for its anti-tumor effects, including lung, breast, nasopharyngeal, colorectal, kidney, pancreatic, prostate, and ovarian cancer, by its molecular implications in cancer metabolism (Reyes-Farias and Carrasco-Pozo, 2019; Shafabakhsh and Asemi, 2019). Licoarylcoumarin and Vestitol, derived from herb licorice, are known to have anti-tumor potential, while the corresponding mechanisms of them remain largely unclear (Hatano et al., 1989; Fukai et al., 2002; Nani et al., 2018). Subsequently, 246 potential targets of ECD and 2406 CRC-related targets were screened, and 140 intersective targets were then identified. Based on the PPI network analysis and four algorithms in Cytoscape software, STAT3, JUN, MAPK3, TP53, MAPK1, RELA, FOS, ESR1, IL6, MAPK14, MYC, and CDKN1A were identified as hub targets in the treatment of CRC. These hub targets were mainly related to tumorigenesis (STAT3, JUN, TP53, CDKN1A, FOS, and MYC), tumor immunity (IL6, RELA), and tumor mechanism (MAPK1, MAPK3, MAPK14, and ESR1) in CRC TME (Bettelli et al., 2005; Zhang et al., 2019; Sun et al., 2020; Stefani et al., 2021). Component-target network was constructed to further present the relationship intuitively between components and corresponding treatment targets and the results of molecular docking demonstrated that the MAPK molecule could be the most valuable and potential therapeutic target of ECD in the treatment of CRC. Consistent with the above findings, the tumorigenesis-, immune-, and metabolism-related pathways were also enriched by GO and KEGG pathway enrichment analysis, indicating MAPK signaling pathway could be the most differential downstream pathway. MAPK (also known as p38), a tumor suppressor, has been highlighted and proven the high susceptibility to tumor development by using genetically modified mouse models (Bulavin et al., 2004; Ventura et al., 2007). And MAPK signaling pathway has also been reported to play a significant role in promoting the development and progression of CRC by affecting cell proliferation and differentiation, blocking the cell cycle, and inhibiting cell apoptosis (Sun et al., 2015). Recently, MAPK inhibitors have been frequently used as cancer therapies, including the direct and indirect effects on TME (Terp et al., 2021). Therefore, combining our results with previous studies, we speculated that ECD might exert its therapeutic effect through the MAPK signaling pathway.

Considering over-reliance on the public datasets and bioinformatics technology could destabilize the results, a systematic experimental validation was performed. In our study, ECD showed certain antiproliferative effects on CRC cells in manners of dose- and time-dependence by cell viability assay, colony formation assay, and EdU incorporation assay. In addition, our results indicated that ECD induced cell cycle arrest in the S phase of CRC cells, down-regulated the transcriptional levels of CDK1 and CDK6, and up-regulated the transcriptional levels of CDKN1A. Cyclin-dependent kinases (CDKs) are reported as cell cycle regulators and the abnormal activation of them can accelerate tumor cell proliferation (Guan et al., 2022). CDKN1A (encoding for p21) is the cell cycle inhibitor and is repressed in the tumors (Peters et al., 2015). A recent study has shown that CDKN1A can also prevent CRC apoptosis by inhibiting the caspase family or the transcriptional repression of pro-apoptotic genes (Li et al., 2018; Nichterwitz et al., 2020). Furthermore, we observed the increasing floating cells and cell debris from the light microscopy images after treatment with ECD, which suggests the pro-apoptosis effects of ECD (Li et al., 2005). Therefore, we further verified the effects of ECD on CRC apoptosis and the results indicated that ECD induced apoptosis of CRC cell lines and downregulated the transcriptional levels of caspase family. In summary, ECD has the anti-proliferative and pro-apoptosis effects on CRC cells and its underlying molecular mechanisms are worth further exploring.

In spite of the fact that ECD has been widely used to treat many diseases in China, very few studies have been conducted to investigate its mechanisms of action. A previous study has shown that ECD provides therapeutic effects on obesity by regulating the gut microbiota in rats (Zhao et al., 2021). The study, performed by our team, has found that one of the potential mechanisms of ECD is regulating the expression of PPARγ and LPL, and some other metabolism-related molecules (Zhang et al., 2020a). Recently, the team of Xi Tan has reported that ECD inhibits the growth of laryngeal carcinoma *via* the STAT3/Cyclin D1 pathway and it was the first study on the mechanism of ECD in the treatment of tumors (Tan et al., 2020). In the current study, based on the results of key targets and pathways analysis, we speculated the underlying molecular mechanisms of ECD in the treatment of CRC are likely to be related to tumor immune and tumor metabolism in TME. Several corresponding pathways, “MAPK signaling pathway,” “p53 signaling,” “PPAR signaling pathway,” and “JAK-STAT signaling pathway” were included, which have been known to be implicated in anti-tumor treatment (Park et al., 2019; Gutting et al., 2021; Nagao et al., 2022). Consistently, the MAPKs, PPARs, STATs, and TP53 were also identified as hub molecules of ECD for treating CRC by the prediction of network pharmacology. We then performed a series of molecular biology experiments to further confirm these findings and all results indicated that ECD exerts anti-tumor effects by regulating the transcription and translation of these hub molecules or pathways in CRC.

In conclusion, based on previous studies, we have explored and supplemented several potential mechanisms of ECD in the treatment of CRC, providing biological evidence for the clinical application of ECD in tumor therapy. In addition, we also found that ECD may be related to the immunotherapy and targeted therapy for CRC based on our results (Figure 5). However, this study has several limitations. First, the effects of ECD on the phosphorylated forms of MAPK and p21 was not explored in this study. Second, we only verified the mechanisms of ECD in the treatment of CRC based on several classical pathways and their hub molecules, and failed to explore novel signaling pathways or targeted molecules. Third, specific mechanisms of ECD and its value in tumor combination therapies still have a long way to go. And we will continue to investigate the further values and mechanisms of ECD and the corresponding combination therapies for CRC treatment in the future.

## Data Availability

The datasets presented in this study can be found in online repositories. The names of the repository/repositories and accession number(s) can be found in the article/Supplementary Material.

## Supplementary material

SUPPLEMENTARY FIGURE S1 The intersective targets between ECD and CRC.

SUPPLEMENTARY FIGURE S2 The light microscopy images of CRC Cells.
