# Supplementary figures and images for: Investigating the effects and mechanisms of Erchen Decoction in the treatment of colorectal cancer by network pharmacology and experimental validation
Source: Front Pharmacol. 2022 Oct 13;13:1000639. doi: 10.3389/fphar.2022.1000639 (PMC9606229; doi:10.3389/fphar.2022.1000639)

**Control**

**ECD (IC25)**

**ECD (IC50)**

**ECD (IC75)**

HT29

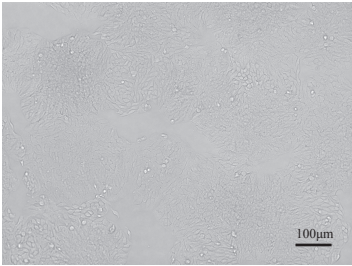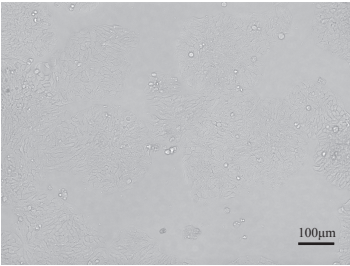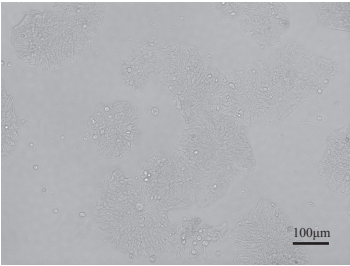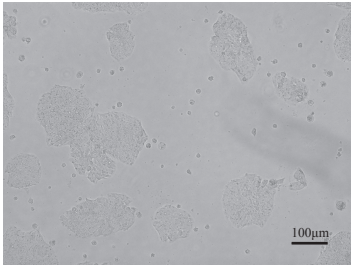

SW620

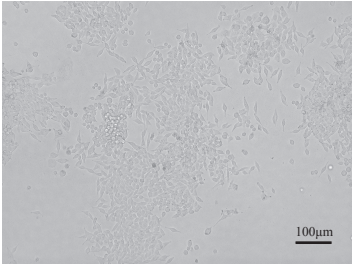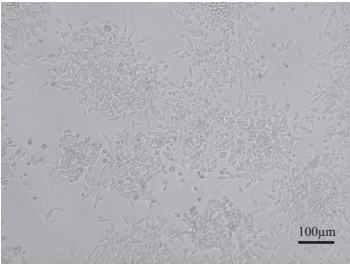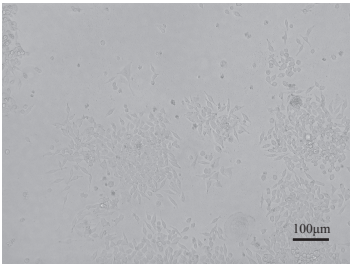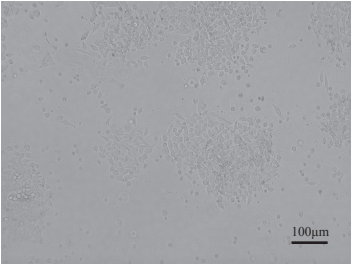

DLD-1

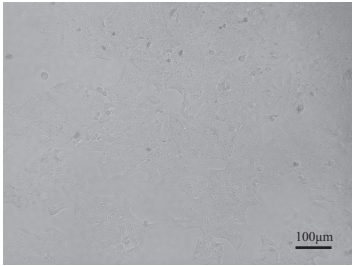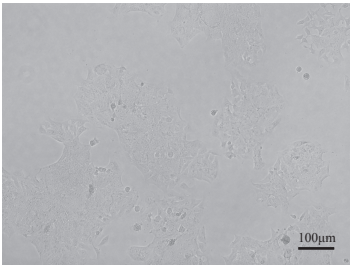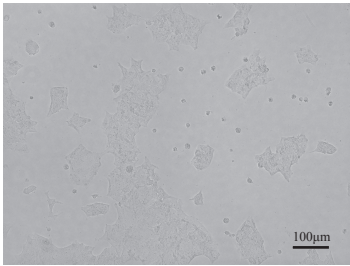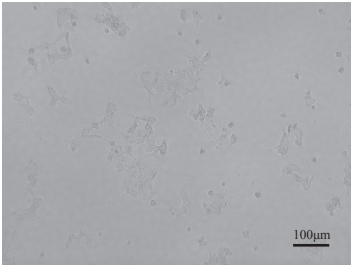

Supplement: Supplementary file 1 [file DataSheet2.PDF]

ECD

CRC

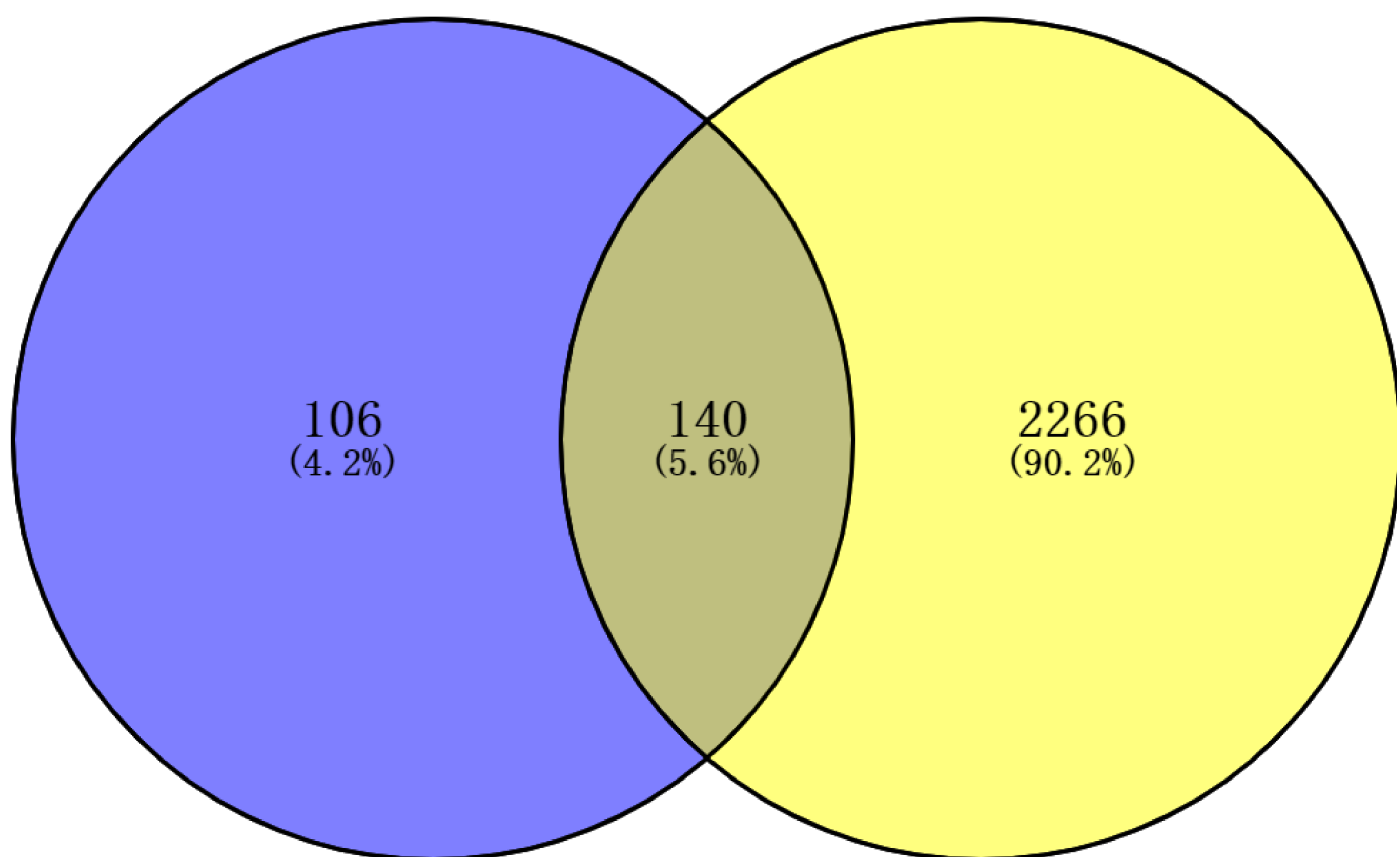

Supplement: Supplementary file 3 [file DataSheet1.PDF]
