# Supplementary material for: Investigating the effects and mechanisms of Erchen Decoction in the treatment of colorectal cancer by network pharmacology and experimental validation
Source: Front Pharmacol. 2022 Oct 13;13:1000639. doi: 10.3389/fphar.2022.1000639 (PMC9606229; doi:10.3389/fphar.2022.1000639)
Supplement: Supplementary file 2 [file Table1.DOCX]

| **Table S1 Real-time PCR primer sequences** | |
| --- | --- |
| Gene | Sequence |
| CDK1 | Forward primer: AAACTACAGGTCAAGTGGTAGCC |
|  | Reversed primer: TCCTGCATAAGCACATCCTGA |
| CDK6 | Forward primer: CCAGATGGCTCTAACCTCAGT |
|  | Reversed primer: AACTTCCACGAAAAAGAGGCTT |
| CDKN1A | Forward primer: CGATGGAACTTCGACTTTGTCA |
|  | Reversed primer: GCACAAGGGTACAAGACAGTG |
| CASP3 | Forward primer: CATGGAAGCGAATCAATGGACT |
|  | Reversed primer: CTGTACCAGACCGAGATGTCA |
| CASP7 | Forward primer: CGGTCCTCGTTTGTACCGTC |
|  | Reversed primer: CGCCCATACCTGTCACTTTATCA |
| CASP8 | Forward primer: AGAGTCTGTGCCCAAATCAAC |
|  | Reversed primer: GCTGCTTCTCTCTTTGCTGAA |
| CASP9 | Forward primer: CTGTCTACGGCACAGATGGAT |
|  | Reversed primer: GGGACTCGTCTTCAGGGGAA |
| MAPK1 | Forward primer: TCACACAGGGTTCCTGACAGA |
|  | Reversed primer: ATGCAGCCTACAGACCAAATATC |
| MAPK3 | Forward primer: CTACACGCAGTTGCAGTACAT |
|  | Reversed primer: CAGCAGGATCTGGATCTCCC |
| MAPK8 | Forward primer: TGTGTGGAATCAAGCACCTTC |
|  | Reversed primer: AGGCGTCATCATAAAACTCGTTC |
| PPARA | Forward primer: ATGGTGGACACGGAAAGCC |
|  | Reversed primer: CGATGGATTGCGAAATCTCTTGG |
| PPARD | Forward primer: ACTGAGTTCGCCAAGAGCATC |
|  | Reversed primer: AGGCGTCATCATAAAACTCGTTC |
| PPARG | Forward primer: TGTGTGGAATCAAGCACCTTC |
|  | Reversed primer: AGGCGTCATCATAAAACTCGTTC |
| STAT1 | Forward primer: ATCAGGCTCAGTCGGGGAATA |
|  | Reversed primer: TGGTCTCGTGTTCTCTGTTCT |
| STAT3 | Forward primer: CAGCAGCTTGACACACGGTA |
|  | Reversed primer: AAACACCAAAGTGGCATGTGA |
| TP53 | Forward primer: GAGGTTGGCTCTGACTGTACC |
|  | Reversed primer: TCCGTCCCAGTAGATTACCAC |
| β-Actin | Forward primer: CTGGGACGACATGGAGAAAA |
|  | Reversed primer: AAGGAAGGCTGGAAGAGTGC |

**Table S2 Full names of the abbreviations in Figure 2C**

| Abbreviation | MOL ID | Active component | Herb name |
| --- | --- | --- | --- |
| GC(Gan cao) |  |  | *Glycyrrhiza glabra* |
| BX(Ban xia) |  |  | *Pinellia ternate* |
| FL(Fu lin) |  |  | *Poria cocos* |
| CP(Chen pi) |  |  | *Citrus reticulata* |
| A1 | MOL000359 | Sitosterol |  |
| B1 | MOL004328 | Naringenin |  |
| GC1 | MOL001484 | Inermine |  |
| GC2 | MOL001792 | DFV |  |
| GC3 | MOL002311 | Glycyrol |  |
| GC4 | MOL000239 | Jaranol |  |
| GC5 | MOL002565 | Medicarpin |  |
| GC6 | MOL000354 | Isorhamnetin |  |
| GC7 | MOL003656 | Lupiwighteone |  |
| GC8 | MOL003896 | 7-Methoxy-2-methyl isoflavone |  |
| GC9 | MOL000392 | Formononetin |  |
| GC10 | MOL000417 | Calycosin |  |
| GC11 | MOL000422 | Kaempferol |  |
| GC12 | MOL004805 | (2S)-2-[4-hydroxy-3-(3-methylbut-2-enyl)phenyl]-8,8-dimethyl-2,3-dihydropyrano[2,3-f]chromen-4-one |  |
| GC13 | MOL004806 | Euchrenone |  |
| GC14 | MOL004808 | Glyasperin B |  |
| GC15 | MOL004810 | Glyasperin F |  |
| GC16 | MOL004811 | Glyasperin C |  |
| GC17 | MOL004814 | Isotrifoliol |  |
| GC18 | MOL004815 | (E)-1-(2,4-dihydroxyphenyl)-3-(2,2-dimethylchromen-6-yl)prop-2-en-1-one |  |
| GC19 | MOL004820 | Kanzonols W |  |
| GC20 | MOL004824 | (2S)-6-(2,4-dihydroxyphenyl)-2-(2-hydroxypropan-2-yl)-4-methoxy-2,3-dihydrofuro[3,2-g]chromen-7-one |  |
| GC21 | MOL004827 | Semilicoisoflavone B |  |
| GC22 | MOL004828 | Glepidotin A |  |
| GC23 | MOL004829 | Glepidotin B |  |
| GC24 | MOL004833 | Phaseolinisoflavan |  |
| GC25 | MOL004835 | Glypallichalcone |  |
| GC26 | MOL004838 | 8-(6-hydroxy-2-benzofuranyl)-2,2-dimethyl-5-chromenol |  |
| GC27 | MOL004841 | Licochalcone B |  |
| GC28 | MOL004848 | licochalcone G |  |
| GC29 | MOL004849 | Licoarylcoumarin |  |
| GC30 | MOL004857 | Gancaonin B |  |
| GC31 | MOL004863 | 3-(3,4-dihydroxyphenyl)-5,7-dihydroxy-8-(3-methylbut-2-enyl)chromone |  |
| GC32 | MOL004864 | 5,7-dihydroxy-3-(4-methoxyphenyl)-8-(3-methylbut-2-enyl)chromone |  |
| GC33 | MOL004866 | 2-(3,4-dihydroxyphenyl)-5,7-dihydroxy-6-(3-methylbut-2-enyl)chromone |  |
| GC34 | MOL004879 | Glycyrin |  |
| GC35 | MOL004882 | Licocoumarone |  |
| GC36 | MOL004883 | Licoisoflavone |  |
| GC37 | MOL004884 | Licoisoflavone B |  |
| GC38 | MOL004885 | Licoisoflavanone |  |
| GC39 | MOL004891 | Shinpterocarpin |  |
| GC40 | MOL004898 | (E)-3-[3,4-dihydroxy-5-(3-methylbut-2-enyl)phenyl]-1-(2,4-dihydroxyphenyl)prop-2-en-1-one |  |
| GC41 | MOL004903 | Liquiritin |  |
| GC42 | MOL004904 | Licopyranocoumarin |  |
| GC43 | MOL004907 | Glyzaglabrin |  |
| GC44 | MOL004908 | Glabridin |  |
| GC45 | MOL004910 | Glabranin |  |
| GC46 | MOL004911 | Glabrene |  |
| GC47 | MOL004912 | Glabrone |  |
| GC48 | MOL004913 | 1,3-dihydroxy-9-methoxy-6-benzofurano[3,2-c]chromenone |  |
| GC49 | MOL004914 | 1,3-dihydroxy-8,9-dimethoxy-6-benzofurano[3,2-c]chromenone |  |
| GC50 | MOL004915 | Eurycarpin A |  |
| GC51 | MOL004924 | (-)-Medicocarpin |  |
| GC52 | MOL004935 | Sigmoidin-B |  |
| GC53 | MOL004941 | (2R)-7-hydroxy-2-(4-hydroxyphenyl)chroman-4-one |  |
| GC54 | MOL004945 | (2S)-7-hydroxy-2-(4-hydroxyphenyl)-8-(3-methylbut-2-enyl)chroman-4-one |  |
| GC55 | MOL004948 | Isoglycyrol |  |
| GC56 | MOL004949 | Isolicoflavonol |  |
| GC57 | MOL004957 | HMO |  |
| GC58 | MOL004959 | 1-Methoxyphaseollidin |  |
| GC59 | MOL004961 | Quercetin der. |  |
| GC60 | MOL004966 | 3'-Hydroxy-4'-O-Methylglabridin |  |
| GC61 | MOL000497 | licochalcone a |  |
| GC62 | MOL004974 | 3'-Methoxyglabridin |  |
| GC63 | MOL004978 | 2-[(3R)-8,8-dimethyl-3,4-dihydro-2H-pyrano[6,5-f]chromen-3-yl]-5-methoxyphenol |  |
| GC64 | MOL004980 | Inflacoumarin A |  |
| GC65 | MOL004985 | Icos-5-enoic acid |  |
| GC66 | MOL004988 | Kanzonol F |  |
| GC67 | MOL004989 | 6-prenylated eriodictyol |  |
| GC68 | MOL004990 | 7,2',4'-trihydroxy－5-methoxy-3－arylcoumarin |  |
| GC69 | MOL004991 | 7-Acetoxy-2-methylisoflavone |  |
| GC70 | MOL004993 | 8-prenylated eriodictyol |  |
| GC71 | MOL004996 | Gadelaidic acid |  |
| GC72 | MOL000500 | Vestitol |  |
| GC73 | MOL005001 | Gancaonin H |  |
| GC74 | MOL005003 | Licoagrocarpin |  |
| GC75 | MOL005007 | Glyasperins M |  |
| GC76 | MOL005008 | Glycyrrhiza flavonol A |  |
| GC77 | MOL005012 | Licoagroisoflavone |  |
| GC78 | MOL005016 | Odoratin |  |
| GC79 | MOL005017 | Phaseol |  |
| GC80 | MOL005018 | Xambioona |  |
| GC81 | MOL005020 | dehydroglyasperins C |  |
| GC82 | MOL000098 | Quercetin |  |
| BX1 | MOL001755 | 24-Ethylcholest-4-en-3-one |  |
| BX2 | MOL002670 | Cavidine |  |
| BX3 | MOL002714 | Baicalein |  |
| BX4 | MOL002776 | Baicalin |  |
| BX5 | MOL000358 | Beta-sitosterol |  |
| BX6 | MOL000449 | Stigmasterol |  |
| BX7 | MOL005030 | Gondoic acid |  |
| BX8 | MOL000519 | Coniferin |  |
| BX9 | MOL006936 | 10,13-eicosadienoic |  |
| BX10 | MOL006957 | (3S,6S)-3-(benzyl)-6-(4-hydroxybenzyl)piperazine-2,5-quinone |  |
| BX11 | MOL003578 | Cycloartenol |  |
| CP1 | MOL005100 | 5,7-dihydroxy-2-(3-hydroxy-4-methoxyphenyl)chroman-4-one |  |
| CP2 | MOL005815 | Citromitin |  |
| CP3 | MOL005828 | Nobiletin |  |
| FL1 | MOL000273 | (2R)-2-[(3S,5R,10S,13R,14R,16R,17R)-3,16-dihydroxy-4,4,10,13,14-pentamethyl-2,3,5,6,12,15,16,17-octahydro-1H-cyclopenta[a]phenanthren-17-yl]-6-methylhept-5-enoic acid |  |
| FL2 | MOL000275 | Trametenolic acid |  |
| FL3 | MOL000279 | Cerevisterol |  |
| FL4 | MOL000282 | Ergosta-7,22E-dien-3beta-ol |  |
| FL5 | MOL000283 | Ergosterol peroxide |  |
| FL6 | MOL000296 | Hederagenin |  |
